# Comparing single-nucleotide polymorphism marker-based and microsatellite marker-based linkage analyses

**DOI:** 10.1186/1471-2156-6-S1-S13

**Published:** 2005-12-30

**Authors:** Ayse Ulgen, Wentian Li

**Affiliations:** 1 G.H. Sergievsky Center, Columbia University, New York, NY, 10032, USA; 2 EMI-0006 INSERM-Université d'Evry, Méthodologie Statistique et Epidémiologie Génétique des Maladies Mulitfactorielles, Evry, 91034, France; 3 The Robert S Boas Center for Genomics and Human Genetics, North Shore LIJ, Institute for Medical Research, Manhasset, NY, 11030, USA

## Abstract

We compared linkage analysis results for an alcoholism trait, ALDX1 (DSM-III-R and Feigner criteria) using a nonparametric linkage analysis method, which takes into account allele sharing among several affected persons, for both microsatellite and single-nucleotide polymorphism (SNP) markers (Affymetrix and Illumina) in the Collaborative Study on the Genetics of Alcoholism (COGA) dataset provided to participants at the Genetic Analysis Workshop 14 (GAW14). The two sets of linkage results from the dense Affymetrix SNP markers and less densely spaced Illumina SNP markers are very similar. The linkage analysis results from microsatellite and SNP markers are generally similar, but the match is not perfect. Strong linkage peaks were found on chromosome 7 in three sets of linkage analyses using both SNP and microsatellite marker data. We also observed that for SNP markers, using the given genetic map and using the map by converting 1 megabase pair (1 Mb) to 1 centimorgan (cM), did not change the linkage results. We recommend the use of the 1 Mb-to-1 cM converted map in a first round of linkage analysis with SNP markers in which map integration is an issue.

## Background

Using single-nucleotide polymorphisms (SNP) for linkage analysis is a relatively new strategy. With only two alleles, the homozygosity rate for SNP markers is high. Homozygous parents are not informative for linkage. SNP-based linkage analyses using a single SNP may not be as successful as linkage analyses using microsatellite markers. However, SNP-based linkage analyses may work better when several neighboring markers are combined together (i.e., multipoint analysis). These SNP combinations can be considered as one composite marker with more alleles [1]. However, due to phase ambiguity, the equivalence between multiple SNPs and a single multi-allele marker is not exact [2].

Even though on a single marker basis SNP markers are not as informative as microsatellite markers, technological advantages have pushed SNP-based linkage analysis forward and SNPs can now be used both for linkage and association studies. This advance is possible because SNPs are relatively inexpensive, have high-throughput production, and dense coverage of the genome. The Affymetrix 10 k chip [3,4], for example, has increasingly been applied to linkage analysis [5].

Comparisons between linkage analyses using SNPs and microsatellite markers have recently been reported in the literature [6]. This work is an addition to this effort, using the COGA (Collaborative Study on the Genetics of Alcoholism) data as part of the Genetic Analysis Workshop 14 (GAW14). We compare nonparametric linkage analysis results obtained from SNP and microsatellite markers. We conducted multipoint analyses and note changes in linkage results when minor details of the map are altered. Given that the multipoint analytic approach is potentially more powerful than single-point analysis for mapping disease loci in the absence of genotyping errors and genetic map misspecifications [7,8], we carry out both single-point and multipoint nonparametric linkage analyses for the microsatellites. We show the genome-wide linkage results in this paper.

## Methods

### Pedigree and marker data

The 143 families (1,614 individuals) were included in the pedigree data, though only roughly 1,300–1,400 people are genotyped with microsatellite markers, Affymetrix SNP, or Illumina SNP markers (the exact number of genotyped samples is given in the papers describing the data provided to GAW participants). The numbers of microsatellite markers, Illumina SNP markers, and Affymetrix SNP markers are 315 (mean spacing 10.316 cM, SD 7.656), 4,596 (mean spacing 0.775 cM, SD 1.173 cM), and 10,805 (mean spacing 0.326 cM, SD 0.615 cM). We used the same set of individuals for the microsatellite marker, Illumina SNP, and Affymetrix SNP genome scans.

### Phenotype definition

The phenotype we used was ALDX1, where affectation includes both DSM-III-R alcohol dependence and Feighner alcohol dependence. We coded an individual as affected if he or she was an alcoholic (ALDX1 = 5), as unaffected if they never drank or if they were unaffected with some symptoms (ALDX1 = 1, 2, 3), and as unknown if there was no information about their symptoms (ALDX1 = 0).

### Computational methods

MERLIN [9] is currently the only program that can handle extremely large numbers of SNP markers in a pedigree linkage analysis. Because only nonparametric linkage (NPL) analysis is implemented in MERLIN program, we compared NPL results, a method which takes into account allele sharing among several affected persons [10]. In the future, as other programs such as dChipLinkage [11] that implement model-based linkage analysis for SNP data become available, comparisons can be extended.

We ran MERLIN twice on the microsatellite markers: once for single-point analysis and again for multipoint analyses. MERLIN was run twice for the Illumina SNP data also. The first time we used a genetic map that was converted from physical (base pair) position information (1 Mb is converted to 1 cM); and the second time we used the genetic map position provided by GAW14, but manually added an arbitrary small distance when two or more SNPs had the same position in centimorgans. MERLIN was run three times for the Affymetrix SNP data: 1) using the genetic map with the 1 Mb-to-1 cM conversion, 2) using the genetic map given by the GAW14, and 3) using the genetic map given by Affymetrix (GC Kennedy, personal communication). We investigated the 1 Mb-to-1 cM map conversion because there may be other cases in which the genetic map information is not available. We were interested to see whether this simple method was successful with the COGA data.

MERLIN (version 0.10.2) was run with 24 bits (a measure of the pedigree complexity), on a computer with 8 gigabytes of memory. Even with this upper memory limit, 9 pedigrees (pedigrees 10008, 10022, 10039, 10052, 10083, 10091, 10104, 10110, 10131) were skipped by MERLIN. We used the same program, and the same individuals in the sample, for all analyses. As a result, the same exact set of 134 families (1,371 individuals) was analyzed in each case. Even with extended computer hardware and a moderately restricted sample, for an average size chromosome, the computing time was in excess of 2 to 3 hours for each SNP dataset. The total computing time for results included in this paper was in the range of 300 hours.

## Results

**Figure 1:**
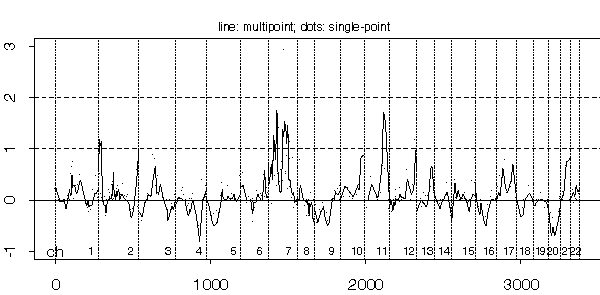
**K-C LOD scores for NPL_all _for microsatellite markers. **Solid lines correspond to multipoint analysis and dots correspond to single-point analysis.

**Figure 2:**
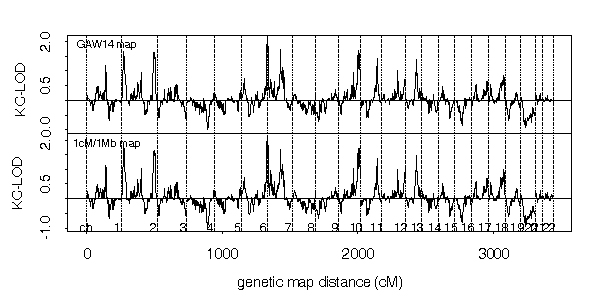
**K-C LOD scores for NPL_all _for the Illumina SNP markers. **Two sets of results are included: one using the genetic map given by GAW14 and another using the map derived from the 1 Mb-to-1 cM rule.

**Figure 3:**
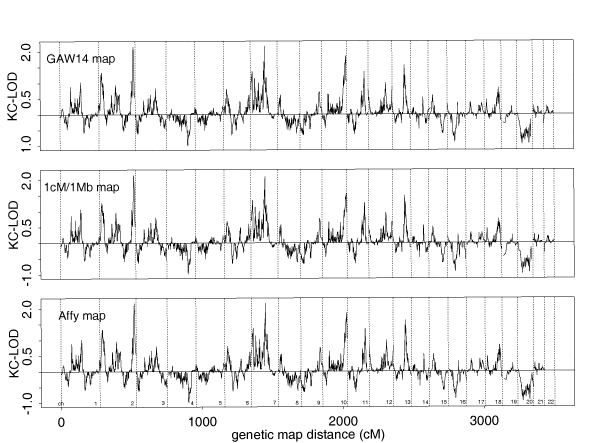
**K-C LOD scores for NPL_all _for the Affymetrix SNP markers. **Three sets of results are included: one using the genetic map given by Affymetrix (G. Kennedy, personal communication), one using the map derived from the 1 Mb-to-1 cM rule, and another using the genetic map given by GAW14.

Figures 1,2,3 show the Kong-Cox LOD score (KC-LOD) [12] for all 22 autosomal chromosomes for microsatellite markers and each of the two SNP sets. The shape of the -log_10_(*p*-value) curve with the *p*-value from the NPL_all _test is very similar to Figures 1,2,3, with the maximum value of -log_10_(*p*-value) roughly equal to the maximum value of LOD plus 1 (the result of *p*-values is not shown).

Figure 1 shows the linkage results for microsatellite markers. Multipoint results are shown with a solid line, and single-point results are plotted using dots. There is a general tendency for the two to move together, but the exact peak heights are different. For single-point analysis, only one marker on chromosome 7 exceeds LOD = 2. For multipoint analysis, only chromosomes 7 and 11 contain LOD > 1 regions.

Figure 2 shows two sets of KC-LOD curves for the Illumina SNP panel. The 1 Mb-to-1 cM map and the one using the given genetic map are shown. The two curves are almost indistinguishable. Only when chromosomes are plotted one by one (not shown) is it possible to see some slight differences between the two curves in some regions. The only region in which the LOD score is greater than 2 is located on chromosome 7, though the left telomeric region of chromosome 2, the right telomeric region of chromosome 6, and the right telomeric region of chromosome 10 also show peaks close to a LOD score of 2.

There are three sets of KC-LOD scores shown in Figure 3 corresponding to the Affymetrix SNP markers. We observe that the differences between the three sets of KC-LOD curves are very small. This is even more striking if we consider the fact that some SNP orders were switched in the Affymetrix map when compared to the map originally provided. Admittedly, this order change was made only for SNPs whose genetic map positions were the same in the map provided. In other words, these SNPs are already close to one another. In Figure 3, chromosomes 2 and 7 show the highest peaks (LOD > 2), followed by chromosomes 10 and 13 (LOD > 1.5).

**Figure 4:**
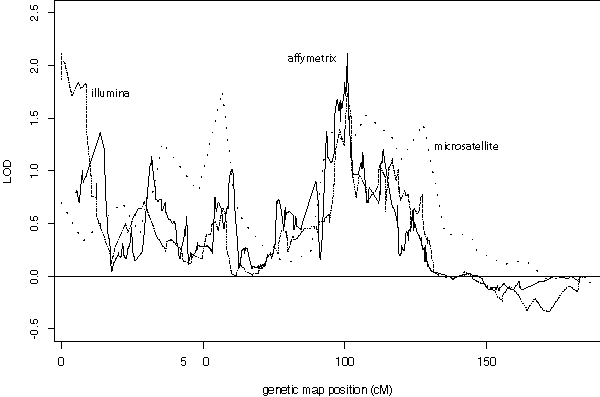
**K-C LOD scores for NPL_all _for three datasets on chromosome 7. **Three linkage analysis results are included: microsatellite multipoint, one run for Illumina data (1 Mb-to-1 cM rule), and one run for Affymetrix data (1 Mb-to-1 cM rule).

To compare linkage analyses from different types of markers, we plot the 3 sets of KC-LOD results for chromosome 7 in Figure 4 (one set for microsatellite markers: multipoint, one set for Illumina SNP markers, and one set for Affymetrix SNP markers). Because the physical locations for microsatellite markers were not provided, the only way to plot all results together is to use the genetic map as the x-axis. For the result based on 1 Mb-to-1 cM genetic maps, we still use the given genetic map for the x-axis to ensure that the same SNP marker is plotted at the same location. It can be seen from Figure 4 that both SNP marker sets lead to more consistent linkage results. One interesting observation is that the Illumina SNP map starts farther left than the Affymetrix map, and the Illumina SNP has much stronger evidence for linkage. The linkage results from microsatellite markers and SNP markers match less well, though the general trend is consistent (e.g., a high peak around 100 cM).

## Discussion and Conclusions

This study addresses two questions: first, are the results consistent when comparing microsatellite marker and SNP-based linkage analyses? Second, for SNP-based linkage analysis, can the 1 Mb-to-1 cM genetic map be used when only the physical location is provided for the SNP markers instead of genetic map distance? The answer to the first question seems to be that the two linkage results match on a crude level, but do not match on a finer level. For all three sets of marker data, the highest linkage peaks appear in chromosome 7. This indicates that on the genome-wide level, microsatellite and SNP markers do lead to consistent results. On the other hand, for intermediate linkage peaks (e.g., LOD ~ 1), three sets of marker data do not match. For example, a peak on chromosome 12 for Illumina and Affymetrix SNP data does not appear in the microsatellite data.

One strategy to explain the discrepancy between the two sets of markers is to examine the information content. Evans et al. [13] simulated data in order to compare SNP versus microsatellite maps. They state that microsatellites may have less information content than SNPs due to the sparse spatial density of microsatellites markers. They also state that parental genotypes maximize the informativeness of sparse microsatellites (a similar conclusion on the importance of parental genotypes for the reconstruction of haplotypes, and thus linkage results, is reached by Li and Gregersen [14]). They conclude that given a density of 1 microsatellite marker per 1 or 2 cM, the information content for microsatellites is close to 100% and there is no point in increasing the density, given that the parents are genotyped. However, they observe that the information content drops to 70% when a sparser map is used. They observe that when the parental genotypes are not available, the information content drops to 70% even with a high density map, which is approximately the same level as a sparse map of microsatellites when parents are genotyped. Furthermore, they conclude that the information content could be as low as 30% when a 1 microsatellite per 10 cM map is used. We note that, in this dataset parental data are relatively complete, which is unusual for most complex diseases, especially late-onset diseases. Given that in our dataset the density of SNPs is much higher (3,000 cM per 10,000 SNPs, i.e., 0.3 cM per SNP, versus 3,000 cM per 300 microsatellites, i.e., 10 cM per microsatellite) and the parental genotypes are relatively complete, we can conclude with confidence that SNPs have higher information content compared with microsatellites, and the linkage result obtained from SNPs in this dataset should be trusted more than the results obtained from the microsatellites.

The answer to the second question seems to be that we can indeed use the simple 1 Mb-to-1 cM rule to generate a genetic map for linkage analysis. Using the 1 Mb-to-1 cM conversion rule is equivalent to the assumption that the recombination rate was homogeneous along a chromosome, which is known to be unrealistic. The reason that this simplistic map is still able to reproduce the linkage result based on a more detailed genetic map is perhaps that the inter-marker distances are relatively short. If we consider Affymetrix's 10 k chip for example, as 10,000 SNP covers the whole human genome with 3000 Mb, the inter-marker distance is on average 300 kb, or roughly 0.3 cM. Even with a large variation in local recombination rate, the range of the absolute value of the inter-SNP distance is small. Our findings showed that the cM/Mb = 1 rule may prove to be a useful tool in situations when the genetic marker map is missing or incomplete.

In conclusion, we have carried out genome-wide NPL multipoint analyses seven times using both microsatellite and Illumina or Affymetrix SNP markers, on the same pedigree data with identical affection status definition. The linkage signals obtained from the Illumina SNP data and Affymetrix SNP data are more similar than those obtained from the microsatellite markers. However, all three datasets point to chromosome 7 as having the strongest linkage signal. We also compare linkage analyses with SNP markers using the given genetic map and using the map derived from the simple rule of 1 Mb-to-1 cM. We showed that the linkage signals obtained from these two genetic maps are essentially the same.

## Abbreviations

COGA: Collaborative Study on the Genetics of Alcoholism

GAW14: Genetic Analysis Workshop 14

KC-LOD: Kong-Cox LOD

NPL: Nonparametric linkage

SNP: Single-nucleotide polymorphisms

## Authors' contributions

AU conducted the MERLIN runs on the microsatellite data, and WL conducted the MERLIN runs on SNP markers. Both authors read and approved the final manuscript.
